# A case of retinitis pigmentosa homozygous for a rare *CNGA1* causal variant

**DOI:** 10.1038/s41598-021-84098-9

**Published:** 2021-02-25

**Authors:** Kohei Saito, Norimoto Gotoh, Inyeop Kang, Toshio Shimada, Takeshi Usui, Chikashi Terao

**Affiliations:** 1 Clinical Research Center, Shizuoka General Hospital, Shizuoka, Japan; 2 Department of Endocrinology, Metabolism and Nephrology, Keio University School of Medicine, Tokyo, Japan; 3 Center for Diabetes, Endocrinology and Metabolism, Shizuoka General Hospital, Shizuoka, Japan; 4 Department of Ophthalmology, Shizuoka General Hospital, Shizuoka, Japan; 5 Fujinomiya Gotoh Eye Clinic, Shizuoka, Japan; 6 Department of Medical Genetics, Shizuoka General Hospital, Shizuoka, Japan; 7 Department of Applied Genetics, School of Pharmaceutical Sciences, University of Shizuoka, Shizuoka, Japan; 8 Laboratory for Statistical and Translational Genetics, RIKEN Center for Integrative Medical Sciences, Kanagawa, Japan; 9 Division of Statistical Analysis, Research Support Center, Shizuoka General Hospital, 4-27-1 Kita Ando, Aoi-Ku, Shizuoka-shi, Shizuoka, 420-8527 Japan

**Keywords:** Genetics, Eye diseases, Retinal diseases

## Abstract

Retinitis pigmentosa (RP) is a heterogenous hereditary disorder leading to blindness. Despite using next-generation sequencing technologies, causal variants in about 60% of RP cases remain unknown. The heterogeneous genetic inheritance pattern makes it difficult to pinpoint causal variants. Besides, rare penetrating variants are hardly observed in general case–control studies. Thus, a family-based analysis, specifically in a consanguineous family, is a clinically and genetically valuable approach for RP. We analyzed a Japanese consanguineous family with a member suffering from RP with a typical autosomal recessive pattern. We sequenced five direct descendants and spouse using Whole-exome sequencing (WES) and Whole-genome sequencing (WGS). We identified a homozygous pathogenic missense variant in *CNGA1* (NM_000087.3, c.839G > A, p.Arg280His) in the proband, while we found no homozygous genotypes in the other family members. *CNGA1* was previously reported to be associated with RP. We confirmed the genotypes by the Sanger sequencing. Additionally, we assessed the homozygous genotype in the proband for the possibility of a founder mutation using homozygosity analysis. Our results suggested the two copies of the variant derived from a founder mutation. In conclusion, we found the homozygotes for c.839G > A in *CNGA1* as causal for RP.

## Introduction

Retinitis pigmentosa (RP) is a genetic disorder causing blindness due to the degeneration of photoreceptor cells. RP has various inheritance patterns, of which 15–25%, 5–10%, and 5–15% are autosomal dominant, autosomal recessive (AR), and X-linked, respectively^1^. There are more than 2 million patients with RP worldwide^2^, and RP is the second cause of visual impairment in Japan^3^. Various treatment approaches have been developed or proposed, including drug treatment such as 9-cis-retinyl acetate^4^, cell transplantation^5^ (stem or retinal cells), and gene therapy^5^ (e.g., targeting to *RPE65* mutations^6^). As the latest treatments can be applied only in confirmed diagnosis cases with specific genetic backgrounds, genetic analysis has become clinically meaningful for RP patients.

Since Next-generation sequencing (NGS) became a standard tool for genetic research of RP^7,8^, more than 70 RP-related genes have been reported to date (Retnet: http://www.sph.uth.tmc.edu/retnet/ June 20, 2020). However, despite the increase of extensive large cohort studies using the NGS, approximately 60% of RP cases' genetic etiology has remained unknown^9^. In addition to a cohort study, a family-based analysis has also been an informative and meaningful approach to exploring genetic etiology. Specifically, the analysis with consanguineous families provides more rigorous assessments of heritability and pathogenicity.

Here, we describe a Japanese consanguineous family showing a typical autosomal recessive pattern of RP (arRP) caused by a homozygous rare *CNGA1* variant (NM_000087.3, c.839G > A, p.Arg280His). Besides, we revealed the variant was in the homozygous identical-by-descent segments, suggesting a founder mutation effect.

## Results

**Figure 1:**
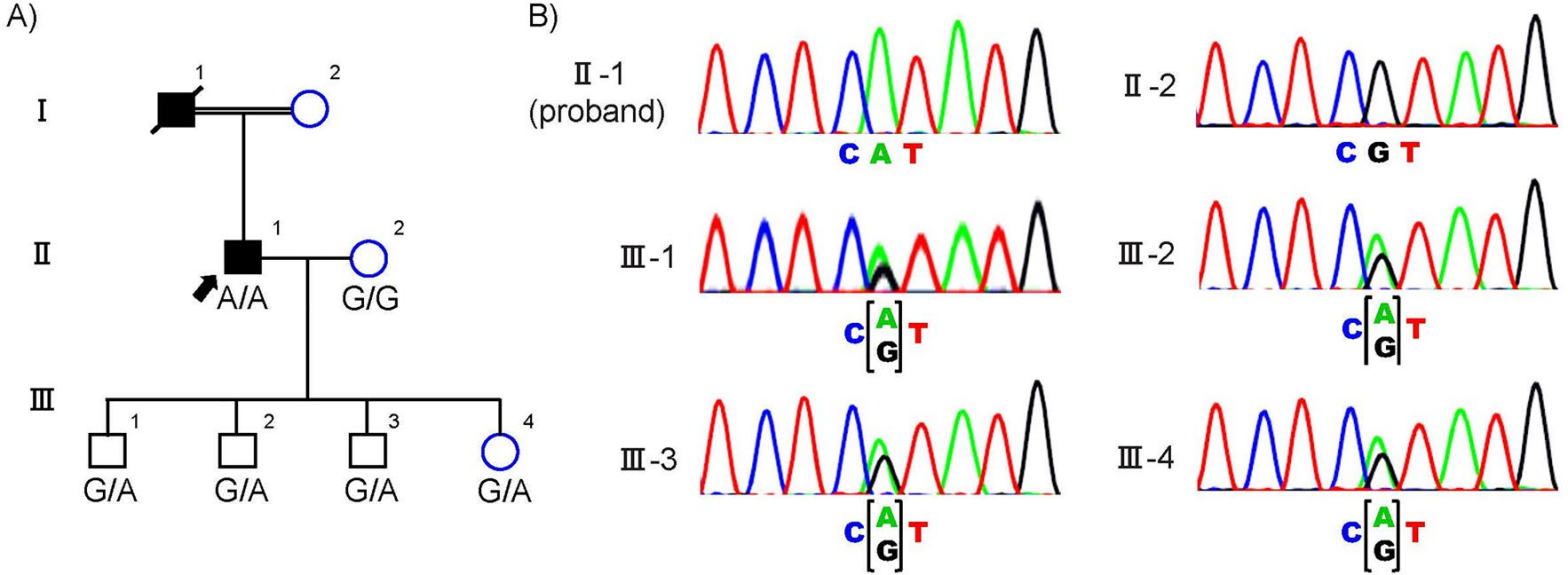
Genetic analysis of the consanguineous family with retinitis pigmentosa. (**A**) Family pedigree with variant in CNGA1. Circles represent females and squares represent males. Black filled squares represent patients with RP. Proband is indicated by black arrow. (**B**) Electropherograms around codon 280 in the CNGA1 gene in the peripheral blood DNA. The result of the proband showed c.839G > A in homozygosity (II-1). The result of his partner showed wild (II-2). The result of the other unaffected members showed c.839G > A in heterozygosity.

The family history showed a typical AR inheritance pattern (Fig. 1). We performed the whole genome sequence (WGS) for the proband (II-1) and the whole exome sequence (WES) for five unaffected members (II-2, III-1, III-2, III-3, III-4). The coverage of the targeted exon site was × 38 and × 106 ± 2.3(mean ± SD) for WGS and WES, respectively.

**Figure 2:**
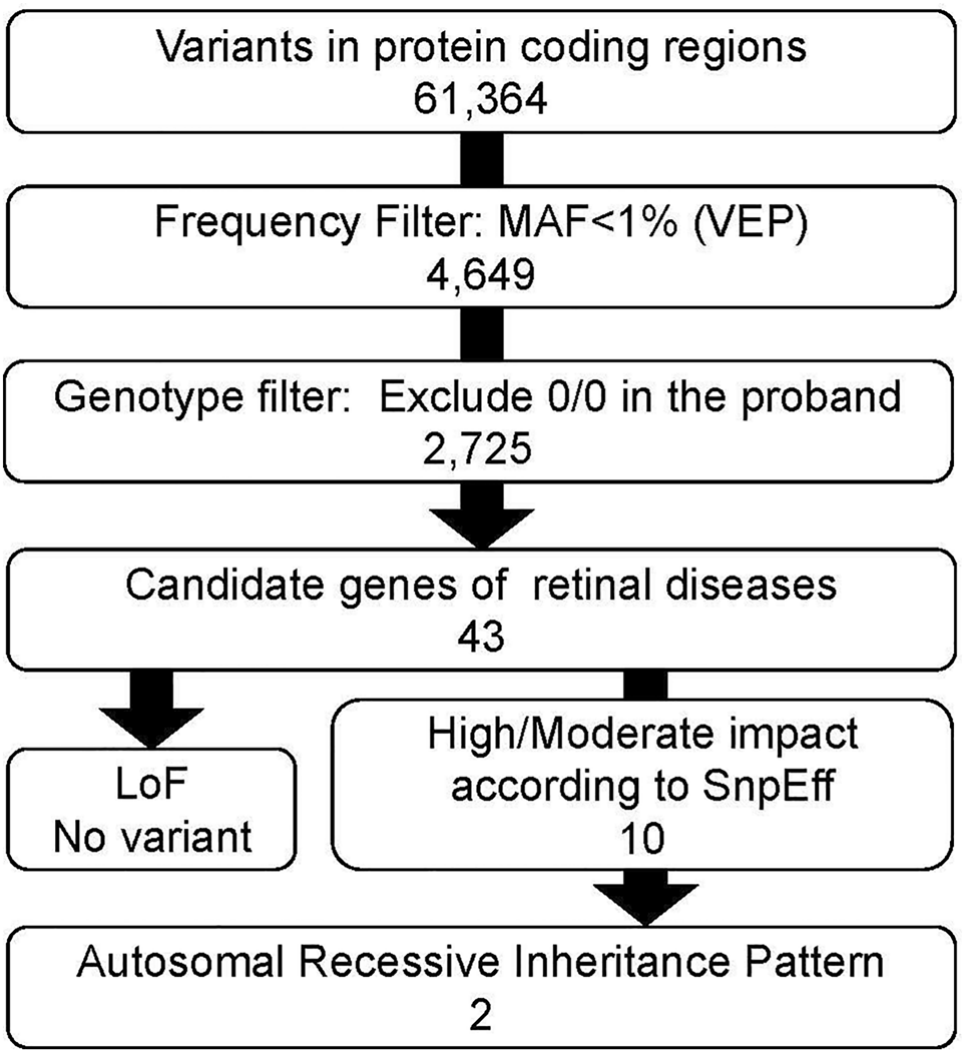
Variant filtering schemes. This figure shows the overview of variant filtering steps. The number shows the sum of variants detected in the jointed data of six family members. *DP* depth, *GQ* genotype quality, *Lof* loss of function, *MAF* minor allele frequency, *VEP* Ensembl Variant Effect Predictor.

After joint calling, we identified a total of 98,841 variants in at least one of the six subjects. Next, we filtered out variants with either DP < 20 or GQ < 20. As a result, 61,634 variants remained including 57,430 Single-Nucleotide Polymorphisms (SNPs), 1906 insertions, and 2301 deletions. Functional annotations revealed 13,331 (21.6%) missense, and 156 (0.25%) nonsense variants. Transition-transversion ratio (Ts/Tv) was 2.6. The Ts/Tv has been used as a QC metric in WES and is expected to above 2.0 within coding regions^10,11^. Supposing a rare pathogenic causal variant in this family, we further filtered variants with a maximum minor allele frequency < 0.01 in 1000 Genomes project^12^, NHLBI Exome Variant Server (https://evs.gs.washington.edu/EVS/), and gnomAD^13^ (Fig. 2). We then excluded variants for which proband’s genotypes contained alternative alleles (i.e., not homozygous for a reference allele, 0/0). After the filtering, the 2725 variants remained. We further restricted candidates of a causal variant in 790 genes related to retinal diseases in the RetNet database (https://sph.uth.edu/retnet/, updated on June 20, 2020), resulting in 43 variants. Since there was no loss of function variants, we focused on the 10 missense variants with the functional impact of “High” or “Moderate”. We could not identify the genotype of the I-1, as we could not obtain the sample. However, we speculate I-1 may have this variant as a homozygous manner due to possible consanguineous marriage of his parents, as the regional characteristics of their living area. Thus, with the consideration of the AR inheritance pattern, we identified homozygous suspected-pathogenic variants in the two genes, namely, the *CNGA1* (NM_000087.3, c.839G > A, p.Arg280His) and *KCNV2* (NM_133497.4, c.1063T > C, p.Phe355Leu). *CNGA1* is a well-known gene associated with RP. *CNGA1* is a well-known gene associated with RP, and according to HDMG (http://www.hgmd.org), the variant was “disease causing” to RP (reported to be disease-causing in the corresponding literature)^9^. The variant in the *KCNV2* was found in the Clinvar database with conflicting interpretations of pathogenicity for non-RP disorders (likely benign for dystrophy with supernormal rod response and uncertain significance for Retinal dystrophy). According to ACMG/AMP guidelines^14^, the variant in the *CNGA1* was classified as pathogenic (Supplemental Table S1), and the variant in the *KCNV2* was classified as Uncertain Significant (Supplemental Table S1). From these annotation analyses, we considered the variant in the CNGA1 as a causal variant for arRP in this family.

Then, to analyze whether the homozygote variants derived from a common ancestor, we performed ROH analysis. As a result, the homozygous regions in the affected proband (II-1) had a total of 31 (129 Mb) homozygous regions that contained the homozygous variants in the *CNGA1*. The unaffected four descendants had no homozygous regions, and the mother had 1 homozygous part on chromosome 3 (3.4 M). These findings confirmed strong consanguinity in the proband and indicated that the homozygous variants in the *CNGA1* derived from a founder mutation.

We performed direct sequencing for the variant in the *CNGA1* in all subjects using the Sanger Sequence. We confirmed that the affected proband was the only one who carried two copies of the variant (Fig. 1).

## Discussion

In the present study, we identified a homozygous disease-causing variant c.839G > A in the *CNGA1* in a consanguineous Japanese family with arRP using NGS sequencing and Sanger sequencing.

Our study showed two important novel points. First, this is the first time to classify the variant in the *CNGA1* (NM_000087.3, c.839G > A, p.Arg280His) as pathogenic according to the ACMG/AMP guideline^14^ (Supplementary Table S1). Although the variant has already been found to be associated with RP in a large sequencing study in a Japanese population^9^, to predict the pathogenicity, the previous study did not follow the ACMG/AMP guideline^14^ but depended on in-silico prediction algorithms and conservation scores. The evaluation of pathogenicity based on the ACMG/AMP guideline is clinically important since clinicians see their patients and diagnose genetic diseases in the clinical settings based on evaluations of variants by the ACMG/AMP guideline. Furthermore, it is essential to validate a variant's pathogenicity in independent samples. Otherwise, a single case with the variant is used to define its pathogenicity, and the definition will affect many potential patients. Second, in our study, we found that the variant in the family of the current analysis derived from a founder mutation.

*CNGA1* is known to be a susceptibility gene to RP, especially arRP. The estimated prevalence of RP with variants in the *CNGA1* is approximately 2–5% with a slight deviation to the Asian population^15–17^. Among the causative gene with arRP, the *CNGA1* was estimated to account for a similar proportion^17^ to the EYS gene (5–16%)^18,19^ The first report of patients with arRP with variants in the *CNGA1* was in 1995^20^. To date, 28 missense/nonsense, 10 small deletion mutations, and 1 splicing substitutions in the *CNGA1* are found in the HDMG database (http://www.hgmd.org). The variant c.839G > A in the *CNGA1* is too rare (allele frequency: 0.0032% from gnomAD) to be found in the general population. Interestingly, this variant has also been identified homozygous in a Japanese RP patient^9^. However, there are no details about clinical and family information, and no functional analysis was conducted other than in silico analysis^9^.

Despite the genetic evidence that the homozygous genotype of this variant leads to RP, its detailed mechanism is still unknown. The *CNGA1* encodes the α subunit of cyclic nucleotide-gated (CNG) channels, one of the cGMP-binding transmembrane channels of cone cells^20^. CNG channel is essential for maintaining the structure and function of photoreceptor cells^21–23^. In the UniProtKB (P29973), there are four known functional domains CNGA1 protein as follows: P-helix (residues 350–360), Selectivity filter (residues 361–369), C-linker (residues 402–484), Cyclic nucleotide-binding domain (residues 485–612) and C-terminal coiled-coil domain (residues 623–666). However, the region containing c.839G > A (p.Arg280His) does not overlap with the functional domains. Therefore, further molecular experiments are necessary to understand the molecular mechanism of this variant.

We found another rare homozygous candidate variant c.1063T > C in the *KCNV2*. The *KCNV2* is known as causing Cone dystrophy retinal 3B (RCD3B)^24^. RCD3B is a rare disease with supernormal rod responses, which is distinct from RP with peripheral retina atrophy^25^. We found that *KCNV2* was also in the homozygous region, which implicated this variant also derived from the same ancestor in spite of unknown functional significance.

In conclusion, we identified a homozygous rare pathogenic variant c.839G > A in *CNGA1* in a consanguineous Japanese family with arRP using NGS sequencing and the Sanger Sequence. Additionally, this is the first study to classify the variant's pathogenicity according to the ACMG/AMP guideline. Our results also suggested the variant c.839G > A derived from a founder mutation. Furthermore, future functional studies are necessary to conclude the effects of the variant in this study as well as the other known pathogenic variants.

## Methods

### Ethics

This study complied with the standards of the Declaration of Helsinki and the ethics committee approved this study of Shizuoka General Hospital, and we obtained written informed consent from all the participants.

### Subject

**Figure 3:**
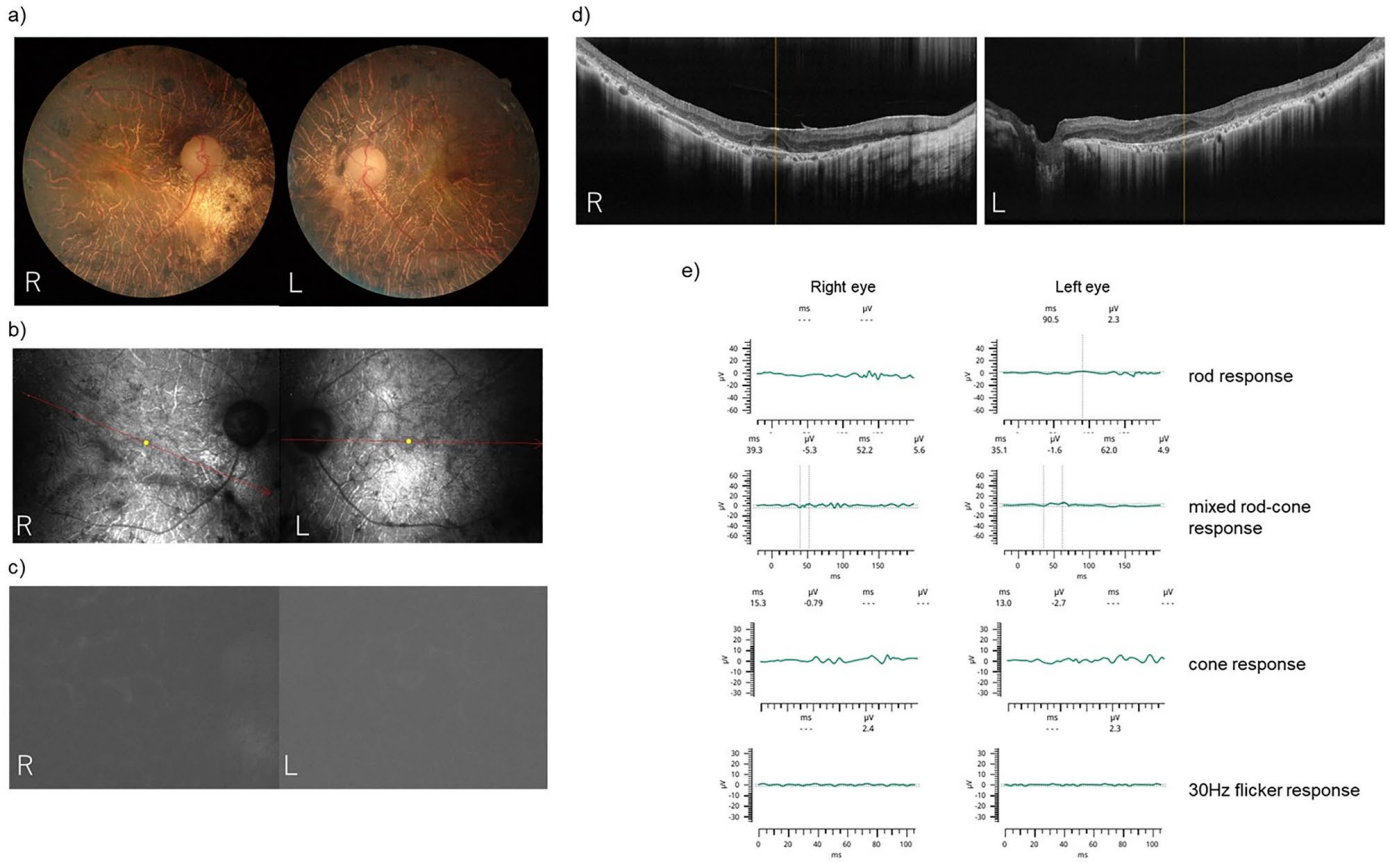
Retinitis pigmentosa phenotype of the proband at 68 years old. (**a**) Color images; (**b**) red free images; (**c**) autofluorescence images; (**d**) macula section with Optical coherence tomography; (**e**) Electroretinography test.

A total of six members participated in this study (Fig. 1). An ophthalmologist diagnosed the proband (II-1) with RP based on comprehensive ophthalmologic examinations, slit-lamp biomicroscopy, color fundus photography, fundus autofluorescence, and ISCEV Standard electroretinogram (The imaging findings of the proband was shown in Fig. 3). All the six participants have performed a fundus examination and electroretinogram. The interviews with the proband determined the affection of the other family members, including their information on daily visual matters. The proband (II-1) is 67 years old. He was diagnosed with RP at the age of 56 and has suffered from visual impairment since then.

### DNA sequence using the Next-generation sequencer

According to the manufacturer's instruction, the genomic DNA of each member was extracted from peripheral blood using PAXgene Blood DNA Kit (Qiagen). The samples were sent to Macrogen Japan Corp. at the University of Kyoto to perform NGS using Novaseq 6000 (Illumina, San Diego, CA, United States) on a 150-bp paired-end read protocol. We conducted the whole-exome sequence (WES), using the SureSelect Human All Exon Kit v6 (Agilent, Santa Clara, CA, United States). We conducted the whole-genome sequence (WGS), using the TruSeq DNA PCR-Free Library Preparation Kit (Illumina, San Diego, CA, United States).

### Data analysis

We performed quality control of fastq files and adapter trimming of the reads, using fastp v0.20.1^26^. In the process of filtering by the fastp, each read was filtered out with >  = 40% of unqualified bases (phred quality <  = Q15) and low complexity (< 30%) (the complexity was the proportion of bases which were different from next ones (base[i] ! = base[i + 1])). In the process of adapter trimming, we trimmed polyG tails (a minimum length was 10). The sequence reads were then aligned to the human GRCh38 reference genome using BWA-MEM (BWA 0.7.17-r1188 package)^27,28^. Duplicates of the reads were marked and removed from the mapped reads using Picard (http://broadinstitute.github.io/picard). We conducted recalibration of base quality scores using GATK 4.1^29^. We performed SNV and indel variant calling separately using the GATK HaplotypeCaller for each sample. We created an interval bed file using the protein-coding region information (Consensus Coding Sequence: CCDS) in the UCSC genome browser (https://genome.ucsc.edu/).

GVCF files were combined and processed with joint-call using GATK (CombineGVCFs and GenotypeGVCFs) based on the family pedigree information. After the joint calling, we performed variant filtering using GATK (VariantFiltration and SelectVariant) with depth > 20 and GQ > 20, followed by Genotype refinement process (CalculateGenotypePosteriors). We annotated variants using SnpEff 4.3t^30^. Information on allele frequency was also added with VEP (Ensembl Variant Effect Predictor)^31^ based on 1000 Genomes project^12^, NHLBI GO Exome Sequencing Project (https://evs.gs.washington.edu/EVS/), and Genome Aggregation Database (gnomAD)^13^. The candidate genes were searched from the RetNet database (https://sph.uth.edu/retnet/). In silico analysis was conducted using SIFT (Dec 6, 2019; http://sift.jcvi.org), PolyPhen-2 (http://genetics.bwh.harvard.edu/pph2), MutationTaster2^32^, and CADD^33^. The ClinVar database (https://www.ncbi.nlm.nih.gov/clinvar/) dbSNP database (https://www.ncbi.nlm.nih.gov/snp/), and Human Gene Mutation Database (HDMG; http://www.hgmd.org) were also referred to obtain functional annotation of the variants.

The runs of homozygosity analysis were performed using PLINK v1.90 (option: homozyg)^34^.

### PCR

To validate genotypes of a pathogenic variant in *CNGA1*, we conducted direct sequencing. A part of exon 11 of *CNGA1* was amplified by PCR using primers of 3′ GGACTGCTGGTAAAGGAAGAACTTAAACTC 5′ for sense primer and 3′ CACCAATGGTAGTCAAAGTCAGTGTAGACC 5′ for antisense primer. The sequencing was carried out by using a sense primer. with a 3500 Genetic Analyzer (Applied Biosystems, Foster City, CA).

## Supplementary Information

Supplementary Information

## Data Availability

The list of variants analyzed in this study is available upon reasonable request to the corresponding author. Data not available due to ethical restrictions.
