# Supplementary material for: A case of retinitis pigmentosa homozygous for a rare CNGA1 causal variant
Source: Sci Rep. 2021 Feb 25;11:4681. doi: 10.1038/s41598-021-84098-9 (PMC7907121; doi:10.1038/s41598-021-84098-9)
Supplement: Supplementary file 1 — Supplementary Information [file 41598_2021_84098_MOESM1_ESM.docx]

**Supplementary file for**

**A case of retinitis pigmentosa homozygous for a rare CNGA1 causal variant**

**Author: Kohei Saito, MD1,2,3; Norimoto Gotoh, MD, PhD1,4,5; Inyeop Kang1,5; Toshio Shimada, MD, PhD1; Takeshi Usui, MD, PhD1,6; Chikashi Terao, MD, PhD1,7,8**

**1Clinical Research Center, Shizuoka General Hospital, Shizuoka, Japan**

**2Department of Endocrinology, Metabolism and Nephrology, Keio University School of Medicine, Tokyo, Japan.**

**3Center for Diabetes, Endocrinology and Metabolism, Shizuoka General Hospital, Shizuoka**

**4Department of Ophthalmology, Shizuoka General Hospital, Shizuoka, Japan**

**5Fujinomiya Gotoh Eye Clinic, Shizuoka, Japan**

**6Department of Medical Genetics, Shizuoka General Hospital, Shizuoka, Japan**

**7Department of Applied Genetics, School of Pharmaceutical Sciences, University of Shizuoka, Shizuoka, Japan.**

**8Laboratory for Statistical and Translational Genetics, RIKEN Center for Integrative Medical Sciences, Kanagawa, Japan.**

**Corresponding author: Chikashi Terao, MD, PhD**

**Division of Statistical Analysis, Research Support Center, Shizuoka General Hospital, 4-27-1 Kita Ando, Aoi-Ku, Shizuoka-shi, Shizuoka 420-8527, Japan**

**Phone: +81-54-247-6111, Fax: +81-54-247-6140**

**Email: chikashi.terao@riken.jp**

**Supplementary Table S1: basic information of each homozygous suspected-pathogenic variants and prediction of their pathogenic effects**

| Gene | CHROM | POS | Reference  SNP | REF | ALT | Reference sequence | MAF | HGVS (DNA) | HGVS (protein) | Consequence | Clin Var | HDMG | SIFT | PolyPhen | Mutation taster | CADD | ACMG  Guideline |
| --- | --- | --- | --- | --- | --- | --- | --- | --- | --- | --- | --- | --- | --- | --- | --- | --- | --- |
| CNGA1 | chr4 | 47937655 | rs375412499 | C | T | NM_000087.3 | <0.01† | c.839G>A | p.Arg280His | missense variant | Uncertain significance | Disease Causing | deleterious  (0) | probably damaging  (0.999) | disease causing | LB(27) | Pathogenic ^§^ |
| KCNV2 | chr9 | 2718802 | rs75645675 | T | C | NM_133497.4 | 0.04‡ | c.1063T>C | p.Phe355Leu | missense variant | Conflicting interpretations of pathogenicity​ B(1);LB(1);Uncertain significance(1) | Not registered | tolerated (0.24) | benign (0.058) | disease causing | LB(15) | Uncertain Significance |

^†^Highest minor allele frequency observed in any population including 1000 Genomes project Phase3, NHLBI GO Exome Sequencing Project, and Genome Aggregation Database

^‡^ Japanese population in 1000 Genomes Phase3

^§^ This is the first time to classify the variant as pathogenic in accordance with the ACMG/AMP guideline^1^ by fulfilling PS4, PM1, PM2, PP2, PP3, and PP4 (without our result, it is interpreted as likely pathogenic with no applicable to PS4)^2^

**References**

1 Richards, S. *et al.* Standards and guidelines for the interpretation of sequence variants: a joint consensus recommendation of the American College of Medical Genetics and Genomics and the Association for Molecular Pathology. *Genet. Med.* **17**, 405-423, doi:10.1038/gim.2015.30 (2015).

2 Kopanos, C. *et al.* VarSome: the human genomic variant search engine. *Bioinformatics* **35**, 1978 (2019).
